# Filament turnover tunes both force generation and dissipation to control long-range flows in a model actomyosin cortex

**DOI:** 10.1371/journal.pcbi.1005811

**Published:** 2017-12-18

**Authors:** William M. McFadden, Patrick M. McCall, Margaret L. Gardel, Edwin M. Munro

**Affiliations:** 1 Biophysical Sciences Program, University of Chicago, Chicago, Illinois, United States of America; 2 Department of Physics, University of Chicago, Chicago, Illinois, United States of America; 3 Institute for Biophysical Dynamics, University of Chicago, Chicago, Illinois, United States of America; 4 James Franck Institute, University of Chicago, Chicago, Illinois, United States of America; 5 Department of Molecular Genetics and Cell Biology, University of Chicago, Chicago, Illinois, United States of America; Northeastern University, UNITED STATES

## Abstract

Actomyosin-based cortical flow is a fundamental engine for cellular morphogenesis. Cortical flows are generated by cross-linked networks of actin filaments and myosin motors, in which active stress produced by motor activity is opposed by passive resistance to network deformation. Continuous flow requires local remodeling through crosslink unbinding and and/or filament disassembly. But how local remodeling tunes stress production and dissipation, and how this in turn shapes long range flow, remains poorly understood. Here, we study a computational model for a cross-linked network with active motors based on minimal requirements for production and dissipation of contractile stress: Asymmetric filament compliance, spatial heterogeneity of motor activity, reversible cross-links and filament turnover. We characterize how the production and dissipation of network stress depend, individually, on cross-link dynamics and filament turnover, and how these dependencies combine to determine overall rates of cortical flow. Our analysis predicts that filament turnover is required to maintain active stress against external resistance and steady state flow in response to external stress. Steady state stress increases with filament lifetime up to a characteristic time *τ*_*m*_, then decreases with lifetime above *τ*_*m*_. Effective viscosity increases with filament lifetime up to a characteristic time *τ*_*c*_, and then becomes independent of filament lifetime and sharply dependent on crosslink dynamics. These individual dependencies of active stress and effective viscosity define multiple regimes of steady state flow. In particular our model predicts that when filament lifetimes are shorter than both *τ*_*c*_ and *τ*_*m*_, the dependencies of effective viscosity and steady state stress on filament turnover cancel one another, such that flow speed is insensitive to filament turnover, and shows a simple dependence on motor activity and crosslink dynamics. These results provide a framework for understanding how animal cells tune cortical flow through local control of network remodeling.

## Author summary

In this paper, we develop and analyze a minimal model for a 2D network of cross-linked actin filaments and myosin motors, representing the cortical cytoskeleton of eukaryotic cells. We implement coarse-grained representations of force production by myosin motors and stress dissipation through an effective cross-link friction and filament turnover. We use this model to characterize how the sustained production of active stress, and the steady dissipation of elastic stress, depend individually on motor activity, effective cross-link friction and filament turnover. Then we combine these results to gain insights into how microscopic network parameters control steady state flow produced by asymmetric distributions of motor activity. Our results provide a framework for understanding how local modulation of microscopic interactions within contractile networks control macroscopic quantities like active stress and effective viscosity to control cortical deformation and flow at cellular scales.

## Introduction

Cortical flow is a fundamental and ubiquitous form of cellular deformation that underlies cell polarization, cell division, cell crawling and multicellular tissue morphogenesis [1–6]. Cortical flows originate within a thin layer of cross-linked actin filaments and myosin motors, called the actomyosin cortex, that lies just beneath the plasma membrane [7]. Local forces produced by bipolar myosin filaments are integrated within cross-linked networks to build macroscopic contractile stress [8–10]. At the same time, cross-linked networks resist deformation and this resistance must be dissipated by network remodeling to allow macroscopic deformation and flow. How force production and dissipation depend on motor activity and network remodeling remains poorly understood.

One successful approach to modeling cortical flow has relied on coarse-grained phenomenological descriptions of actomyosin networks as active fluids, whose motions are driven by gradients of active contractile stress and opposed by an effectively viscous resistance [11]. In these models, spatial variation in active stress is typically assumed to reflect spatial variation in motor activity and force transmission [12], while effective viscosity is assumed to reflect the internal dissipation of elastic resistance due to local remodeling of filaments and/or cross-links [7, 13]. Models combining an active fluid description with simple kinetics for network assembly and disassembly, can successfully reproduce the spatiotemporal dynamics of cortical flow observed during polarization [11], cell division [14, 15], cell motility [16, 17] and tissue morphogenesis [18]. However, it remains a challenge to connect this coarse-grained description of cortical flow to the microscopic origins of force generation and dissipation within cross-linked actomyosin networks.

Studies in living cells reveal fluid-like stress relaxation on timescales of 10-100s [1, 2, 11, 19–21], which is thought to arise through a combination of cross link unbinding and actin filament turnover [7, 13, 22]. Theoretical [23, 24] and computational [25–27] studies reveal that cross-link unbinding can endow actin networks with complex time-dependent viscoelasticity. However, while cross-link unbinding is sufficient for viscous relaxation (creep) on very long timescales *in vitro*, it is unlikely to account for the rapid cortical deformation and flow observed in living cells [26, 28–31]. Experimental studies in living cells reveal rapid turnover of cortical actin filaments on timescales comparable to stress relaxation (10-100s) [32–35]. Perturbing turnover can lead to changes in cortical mechanics and in the rates and patterns of cortical flow [33, 36]. However, the specific contributions of actin turnover to stress relaxation and how these depend on network architecture remain unclear.

Recent work has also begun to reveal mechanisms for active stress generation in disordered actomyosin networks. Theoretical studies suggest that spatial heterogeneity in motor activity along individual filaments, and asymmetrical filament compliance (stiffer in extension than in compression), are sufficient for macroscopic contraction [37, 38], although other routes to contractility may also exist [38]. Local interactions among actin filaments and myosin motors are sufficient to drive macroscopic contraction of disordered networks *in vitro* [39], and the kinematics of contraction observed in these studies support a mechanism based on asymmetrical filament compliance and filament buckling. However, in these studies, the filaments were preassembled and network contraction was transient, because of irreversible network collapse [40], or buildup of elastic resistance [41], or because network rearrangements (polarity sorting) dissipate the potential to generate contractile force [42–45]. This suggests that network turnover may play essential role(s) in allowing sustained production of contractile force. Recent theoretical and modeling studies have begun to explore how this might work [46–48], and to explore dynamic behaviors that can emerge when contractile material undergoes turnover [15, 49]. However, it remains a challenge to understand how force production and dissipation depend individually on the local interplay of network architecture, motor activity and filament turnover, and how these dependencies combine to mediate tunable control of long range cortical flow.

Here, we construct and analyze a simple computational model that bridges between the microscopic description of cross-linked actomyosin networks and the coarse grained description of an active fluid. We represent actin filaments as simple springs with asymmetric compliance; we represent dynamic binding/unbinding of elastic cross-links as molecular friction [50–52] at filament crossover points; we represent motor activity as force coupling on a subset of filament cross-over points with a simple linear force/velocity relationship [53]. Finally, we model filament turnover by allowing entire filaments to appear with a fixed probability per unit area and disappear with fixed probabilities per unit time. We use this model to characterize: first the passive response of a cross-linked network to externally applied stress, then the buildup and maintenance of active stress against an external resistance, and finally the steady state flows produced by an asymmetric distribution of active motors in which active stress and passive resistance are dynamically balanced across the network. Our results reveal how network remodeling can tune cortical flow through simultaneous effects on active force generation and passive resistance to network deformation.

## Models

Our goal is to construct a minimal model that is sufficiently detailed to capture essential microscopic features of cross-linked actomyosin networks (actin filaments with asymmetric compliance, dynamic cross-links, active motors and and continuous filament turnover), but simple enough to explore, systematically, how these microscopic features control macroscopic deformation and flow. We focus on 2D networks because they capture a reasonable approximation of the quasi-2D cortical actomyosin networks that govern flow and deformation in many eukaryotic cells [11, 54], or the quasi-2D networks studied recently *in vitro* [39, 55].

**Fig 1:**
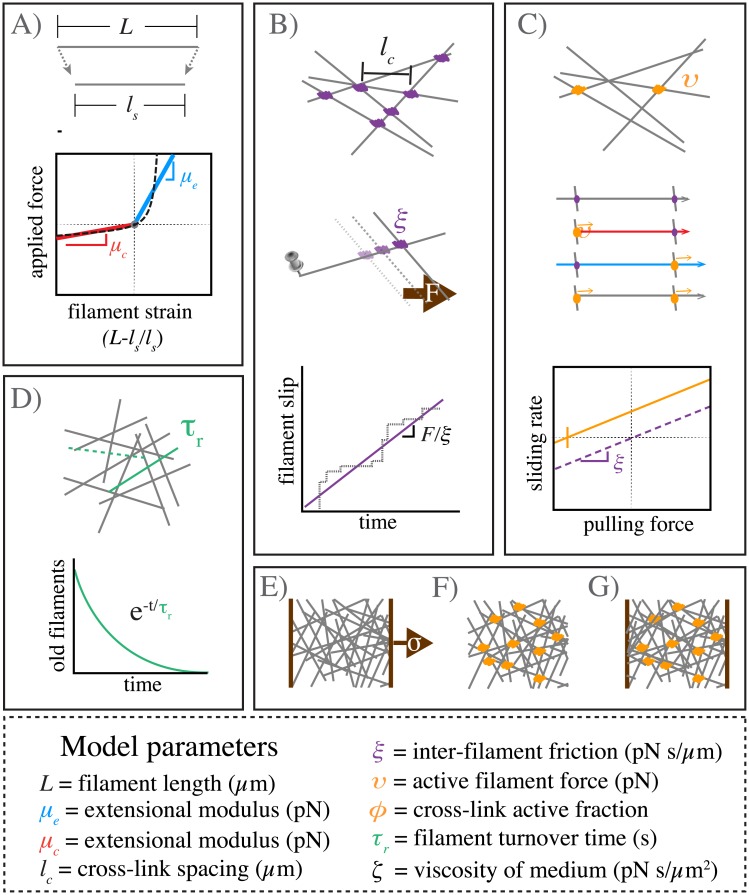
Schematic overview of modeling framework and assumptions. **A)** Filaments are oriented linear springs that are stiffer in extension than in compression. **B)** Cross-linking occurs at all filament crossings; we represent cross link resistance as an effective drag, proportional to the relative velocity of the overlapping filaments. **C)** We represent motor activity as a linear force-velocity relationship with a fixed force at zero velocity directed towards a filament’s pointed (-) end. We implement spatial heterogeneity by imposing motor activity at a fixed fraction of filament crossover points, resulting in variation in the magnitudes of compressive vs extensile vs translational forces along individual filament segments. **D)** Whole filaments disappear at a constant rate; new filaments appear with random positions and orientations at the constant rate per unit area, such that entire network refreshes on a characteristic timescale *τ*_*r*_. **E-G)** Three different simulation scenarios: **E)** Passive response to uniaxial stress, **F)** Free contraction of an active network and **G)** Isometric contraction against a fixed boundary.

Fig 1 provides a schematic overview of our model’s assumptions. We model each filament as an oriented elastic spring with rest length *L*. If index i enumerates over all filaments, then the state of a filament i is defined by the positions of its endpoints **b**_**i**_ and **p**_**i**_, marking its barbed (+) and pointed (-) ends respectively. We define $\hat{u_{i}}$ to be the unit vector oriented along filament i towards its barbed end.

### Asymmetric filament compliance

We assume (Fig 1A) that local deformation of filament i gives rise to equal and opposite elastic restoring forces on its endpoints:

$$\begin{array}{c} F_{p,i}^{\text{elas}}=\mu \gamma_{i}\hat{u_{i}}\text{,}\;F_{b,i}^{\text{elas}}=-F_{p,i}^{\text{elas}} \end{array}$$ (1)

where *γ*_*i*_ = (|**b**_**i**_ − **p**_**i**_| − *L*)/*L* is the strain on filament i, and *μ* is a normalized spring constant. To model asymmetric filament compliance, we use a piecewise linear approximation to the non-linear entropic force extension curve for semi-flexible polymers with lengths less than the persistence length [56, 57]. We set *μ* = *μ*_*e*_ if the strain is positive (extension), and *μ* = *μ*_*c*_ if the strain is negative (compression).

### Drag-like coupling between overlapping filaments

Previous models have represented cross-linkers as elastic connections between pairs of points on neighboring filaments that bind and unbind with either fixed or force-dependent probabilities [25, 58]. Here, we introduce a coarse-grained representation of crosslink dynamics by introducing an effective drag force that couples every pair of overlapping filaments, and which represents a molecular friction arising from the time-averaged contributions of many individual transient crosslinks (Fig 1B). This is a reasonable approximation if all pairs of overlapping filaments have equal access to a non-limiting pool of cross links, and if the rate at which filaments move past one another is slow relative to the unbinding rate of individual crosslinks [59]. This coarse-grained approach has been used to model frictional forces arising from ionic cross-linking of actin filaments *in vitro* [60, 61], and simple force-velocity relationships for systems of cytoskeletal filaments and cross-linking motors [53, 62–64].

To implement coupling through effective drag, for any pair of overlapping filaments i and j, we write the drag force on filament i as:

$$\begin{array}{c} F_{i,j}^{\xi }=-\xi \left(v_{i}-v_{j}\right) \end{array}$$ (2)

where *ξ* is the drag coefficient and **v**_**i**_, **v**_**j**_ are the average centroid velocities of filaments i and j. We apportion this drag force to the two endpoints **p**_**i**_ and **b**_**i**_ of filament i as follows: If **x**_**i**,**j**_ is the position of the filament overlap, then we define **λ**_**i**,**j**_ = |**x**_**i**,**j**_ − **p**_**i**_|/|**b**_**i**_ − **p**_**i**_| to be the fractional position of the overlap point along filament i, and we assign $\left(1-\lambda_{i,j}\right)F_{i,j}^{\xi }$ to endpoint **p**_**i**_ and $\lambda_{i,j}F_{i,j}^{\xi }$ to endpoint **b**_**i**_.

The total crosslink coupling forces on endpoints **p**_**i**_ and **b**_**i**_, due to overlaps along filament i, can then be written:

$$\begin{array}{c} \begin{array}{c} F_{p,i}^{\text{xl}}=\sum_{j}\left(1-\lambda_{i,j}\right)F_{i,j}^{\xi }\\ F_{b,i}^{\text{xl}}=\sum_{j}\;\lambda_{i,j}F_{i,j}^{\xi } \end{array} \end{array}$$ (3)

where the sums are taken over all filaments j that overlap with filament i.

This model assumes a linear relation between the drag force and the velocity difference between attached filaments. Although non-linearities can arise through force dependent detachment kinetics and/or non-linear force extension of cross-links, we assume here that these non-linear effects are of second or higher order.

### Active coupling for motor driven filament interactions

We add motor activity at the point of overlap between two filaments i and j as follows: For each filament in the pair, we impose an additional force of magnitude *υ*, directed towards its pointed (-) end (Fig 1C):

$$\begin{array}{c} F_{i}^{\upsilon }=-\upsilon \hat{u_{i}} \end{array}$$ (4)

We impose an equal and opposite force on its overlapping partner. We distribute these forces to filament endpoints as described above for crosslink coupling forces. Thus, the total force on endpoints i and i+1 due to motor activity on overlap points between filaments i and j can be written as:

$$\begin{array}{c} \begin{array}{c} F_{p,i}^{\text{motor}}=\sum_{j}\left(1-\lambda_{i,j}\right)\;(F_{i}^{\upsilon }-F_{j}^{\upsilon })\;q_{i,j}=\upsilon \;\sum_{j}\left(1-\lambda_{i,j}\right)\;(\hat{u_{j}}-\hat{u_{i}})\;q_{i,j}\\ F_{b,i}^{\text{motor}}=\upsilon \;\sum_{j}\left(\lambda_{i,j}\right)\;(\hat{u_{j}}-\hat{u_{i}})\;q_{i,j} \end{array} \end{array}$$ (5)

where j enumerates over all filaments j that overlap with filament i, and *q*_*i*,*j*_ equals 0 or 1 depending on whether there is an “active” motor at this location. To model dispersion of motor activity, we set *q*_*i*,*j*_ = 1 on a randomly selected subset of filament overlaps, such that $\bar{q}=\phi$, where $\bar{q}$ indicates the mean of *q* (Fig 1C).

### Equations of motion

To write the full equation of motion for a network of actively and passively coupled elastic filaments, we assume the low Reynold’s number limit in which inertial forces can be neglected, and we equate the sum of all forces acting on all filament endpoints to zero to obtain:

$$\begin{array}{c} \begin{array}{c} 0=-\zeta v_{i}^{p}L/2-F_{p,i}^{\text{xl}}+F_{p,i}^{\text{elas}}+F_{p,i}^{\text{motor}}\\ 0=-\zeta v_{i}^{b}L/2-F_{b,i}^{\text{xl}}+F_{b,i}^{\text{elas}}+F_{b,i}^{\text{motor}} \end{array} \end{array}$$ (6)

where the first terms in each equation represent the hydrodynamic drag on the half-filaments adjoining endpoints **p**_**i**_ or **b**_**i**_ with respect to motion at velocities $v_{i}^{p}$ or $v_{i}^{b}$ against the surrounding fluid, and *ζ* is the drag coefficient.

We used a mikado model approach [65] to initialize a minimal network of overlapping unstressed linear filaments in a rectangular 2D domain. We generate individual filaments by laying down straight lines, of length L, with random position and orientation. We define the density using the average distance between cross-links along a filament, *l*_*c*_. A simple geometrical argument can then be used to derive the number of filaments filling a domain as a function of *L* and *l*_*c*_ [66]. Here, we use the approximation that the number of filaments needed to tile a rectangular domain of size *D*_*x*_ × *D*_*y*_ is 2*D*_*x*_*D*_*y*_/*Ll*_*c*_, and that the length density is therefore simply, 2/*l*_*c*_.

Although we do not model thermal forces explicitly, the contribution of thermal fluctuations to filament elasticity are embedded in our coarse-grained representation of asymmetrical filament compliance. In principle, thermally-driven transverse fluctuations of filament segments between crosslink points could influence crosslink binding kinetics. However, for the network mesh sizes considered here, *l*_*c*_ <= 0.5*μm*, the root mean square amplitude of these fluctuations is predicted to be < 5*nm* (see e.g [56]), suggesting that these effects will be minor. Hence, we have chosen to ignore them here.

### Modeling filament turnover

In living cells, actin filament assembly is governed by multiple factors that control filament nucleation, branching and elongation. Likewise filament disassembly is governed by multiple factors that promote filament severing and monomer dissociation at filament ends. Here, we implement a very simple model for filament turnover in which entire filaments appear with a fixed rate per unit area, *k*_*app*_ and disappear at a rate *k*_*diss*_*ρ*, where *ρ* is the filament density (Fig 1D). With this assumption, in the absence of network deformation, the density of filaments will equilibrate to a steady state density, *k*_*app*_/*k*_*diss*_, with time constant *τ*_*r*_ = 1/*k*_*diss*_. In deforming networks, filament density will also decrease under extensional strain and increase under compressional strain. Thus filament density will be set by a dynamic interplay of deformation and density equilibration via turnover (see below and (S1 Appendix A.3)). To implement this model, at fixed time intervals *τ*_*s*_ < 0.01 ⋅ *τ*_*r*_ (i.e. 1% of the equilibration time), we selected a fraction, *τ*_*s*_/*τ*_*r*_, of existing filaments (i.e. less than 1% of the total filaments) for degradation. We then generated a fixed number of new unstrained filaments *k*_*app*_*τ*_*s*_*D*_*x*_*D*_*y*_ at random positions and orientations within the original domain. We refer to *k*_*diss*_ = 1/*τ*_*r*_ as the turnover rate, and to *τ*_*r*_ as the turnover time.

### Simulation methods

Further details regarding our simulation approach and references to our code can be found in the Supplementary Information (S1 Appendix A.1). Briefly, eqs 1–6 define a coupled system of ordinary differential equations that can be written in the form:

$$\begin{array}{c} A\cdot \dot{x}=f\left(x\right) \end{array}$$ (7)

where **x** is a vector of filament endpoint positions, $\dot{x}$ the endpoint velocities, **A** is a matrix with constant coefficients that represent crosslink coupling forces between overlapping filaments, and **f**(**x**) represents the active (motor) and elastic forces on filament endpoints. We smoothed all filament interactions, force fields, and constraints linearly over small regions such that the equations contained no sharp discontinuities. We used a fourth-order Runga-Kutta method to numerically integrate this system of equations to find the time evolution of the positions of all filament endpoints. We generate a network of filaments with random positions and orientations as described above within a domain of size *D*_*x*_ by *D*_*y*_. For all simulations, we imposed periodic boundaries in the y-dimension. To impose an extensional force per unit length (2D stress) on the network, we constrained all filament endpoints within a fixed distance 0.05 ⋅ *D*_*x*_ from the left edge of the domain to be non-moving, then we imposed a rightwards force on all endpoints within a distance 0.05 ⋅ *D*_*x*_ from the right edge of the domain, such that the force per unit length of boundary equals the desired stress value. To simulate free contraction, we removed all constraints at domain boundaries; to assess buildup and maintenance of contractile stress under isometric conditions, we used periodic boundary conditions in both *x* and *y* dimensions.

### Measuring stress, strain, and strain rate

**Fig 2:**
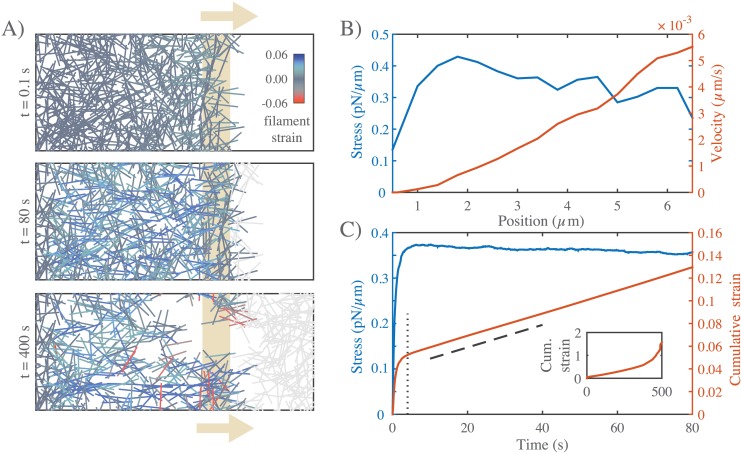
Networks with passive cross-links and no filament turnover undergo three stages of deformation in response to an extensional stress. **A)** Three successive time points from a simulation of a 6.6 × 4 *μm* network deforming under an applied stress of 0.5 *pN*/*μm*. Stress (tan arrows) is applied to filaments in the region indicated by the tan bar. In this and all subsequent figures, filaments are color-coded with respect to state of strain (blue = tension, red = compression). Network parameters: *L* = 1 *μm*, *l*_*c*_ = 0.3 *μm*, *ξ* = 10 *nN* ⋅ *s*/*μm*. **B)** Mean filament stress and velocity profiles for the network in (A) at t = 88s. Note that the stress is nearly constant and the velocity is nearly linear as predicted for a viscous fluid under extension. **C)** Plots of the mean stress and cumulative network strain vs time for the simulation in (B), illustrating the three stages of deformation: (i) A fast initial deformation accompanies rapid buildup of internal network stress; (ii) after a characteristic time *τ*_*c*_ (indicated by vertical dotted line) the network deforms at a constant rate, i.e. with a constant effective viscosity, *η*_*c*_, given by the slope of the dashed line; (iii) at long times, decrease in filament density, and loss of network connectivity, leads to material failure (see inset).

In our 2D model, we measure stress as a force per unit length. We measured the internal network stress at each axial position by summing the axial (x) component of the tensions on all filaments intersecting that position, and dividing by network height *D*_*y*_. We quantified two different forms of strain: the average filament strain, which measures the deformations of individual filaments, and the cumulative network strain, a normalized measure of network deformation defined as the change in axial length of a patch of network divided by its original length. These two measures can differ because filaments can slide relative to one another during deformation and because strained filaments are replaced by unstrained filaments during network turnover. We measured the strain on individual filaments as defined above from *γ*_*i*_ = (|**b**_**i**_ − **p**_**i**_| − *L*)/*L*. Then we averaged this measurement over all filaments in a network to obtain an average filament strain. To measure the average network strain rate, we first measured the mean velocity v(X) at position X (relative to the network boundary at x = 0) to be the average velocities of all filaments intersecting that position. In the cases where we measure network strain or strain rates, we observed an approximately linear dependence of v(X) on X; hence the strain rate is approximately uniform across the network (Fig 2B and S6 Fig). Accordingly, for each filament, we took $\frac{1}{X}\frac{dX}{dt}$ to be an estimate of the strain rate on the network between x = 0 and x = X. We averaged this estimate over all filaments in a domain to get an average strain rate. Finally, to estimate the cumulative network strain at a given time T in the simulation, we integrated the strain rate with respect to time for t = 0 to T.

We assigned biological plausible reference values for all parameters (See Table 1). For individual analyses, we sampled the ranges of parameter values around these reference values shown in S1 Table.

**Table 1:** Simulation parameters with reference values.

Parameter	Symbol	Reference Value
extensional spring constant	*μ*_*e*_	100*pN*
compressional spring constant	*μ*_*c*_	1*pN*
cross-link drag coefficient	*ξ*	$100\frac{pNs}{\mu m}$
solvent drag coefficient	*ζ*	$0.05\frac{pNs}{\mu m^{2}}$
filament length	L	5*μm*
cross-link spacing	*l*_*c*_	0.5*μm*
active filament force	*υ*	10*pN*
active cross-link fraction	*ϕ*	0.1–0.9
domain size	*D*_*x*_ × *D*_*y*_	50 × 20*μm*

## Results

The goal of this study is to understand how cortical flow is shaped by the simultaneous dependencies of active stress and effective viscosity on filament turnover, crosslink drag and on “network parameters” that control filament density, elasticity and motor activity. We approach this in three steps: First, we analyze the passive deformation of a cross-linked network in response to an externally applied stress; we identify regimes in which the network response is effectively viscous and characterize the dependence of effective viscosity on network parameters and filament turnover. Second, we analyze the buildup and dissipation of active stress in cross-linked networks with active motors, as they contract against an external resistance; we identify conditions under which the network can produce sustained stress at steady state, and characterize how steady state stress depends on network parameters and filament turnover. Finally, we confirm that the dependencies of active stress and effective viscosity on network parameters and filament turnover are sufficient to predict the dynamics of networks undergoing steady state flow in response to spatial gradients of motor activity.

### Filament turnover allows and tunes effectively viscous steady state flow

#### Networks with passive cross-links and no filament turnover undergo three stages of deformation in response to an extensional force

To characterize the passive response of a cross-linked filament network without filament turnover, we simulated a simple uniaxial strain experiment in which we pinned the network at one end, and imposed an external stress at the opposite end (Fig 1E). We quantified the internal network stress, the average filament strain, and the cumulative network strain as functions of time (see section entitled Measuring stress, strain, and strain rate above). The typical response to axial stress occurred in three qualitatively distinct phases (Fig 2A and 2C). At short times (up to vertical dotted line in Fig 2C), the network response was viscoelastic, with a rapid buildup of internal stress and a rapid ∼exponential approach to a level of cumulative network strain that represents the elastic limit predicted for a network with rigid irreversible crosslinks [66] S1A Fig). At intermediate times (beyond vertical dotted line in Fig 2C), the local stress and strain rate were approximately constant across the network (Fig 2B), and the temporal response was effectively viscous; internal stress (blue curve in Fig 2C) (and thus filament strain) remained constant, while the cumulative network strain (red curve in Fig 2C) increased linearly with time (dashed line in Fig 2C), as filaments slip past one another against the effective cross-link drag. The linear increase in cumulative network strain corresponds to a nearly constant strain rate. Thus at intermediate times, we can quantify effective viscosity, *η*_*c*_, as the ratio of applied stress to the measured strain rate. Finally, at long times, as cumulative network strain increased, there was a corresponding decrease in filament density. As cumulative network strain approached a critical value (∼ 30% for the simulation in Fig 2), the decrease in filament density lead to decreased network connectivity, local tearing, and rapid acceleration of the network deformation (see inset in Fig 2C).

#### Network architecture sets the rate and timescales of deformation

**Fig 3:**
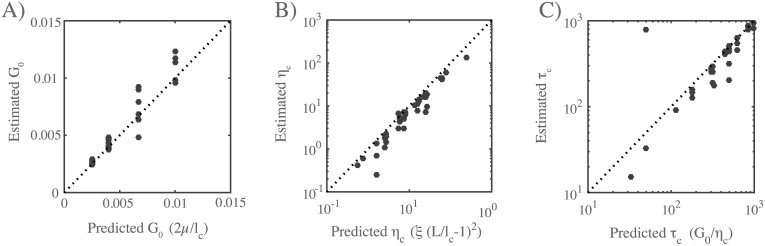
Network architecture sets the rate and timescales of deformation. **(A-C)** Comparison of predicted and simulated values for: **A)** the bulk elastic modulus *G*_0_, **B)** the effective viscosity *η*_*c*_ and **C)** the timescale for transition from viscoelastic to viscous behavior *τ*_*c*_, given by the ratio of the bulk elastic modulus *G*_0_ to effective viscosity, *η*_*c*_. Dotted lines indicates the relationships predicted by theory.

To characterize how effective viscosity and the timescale for transition to effectively viscous behavior depend on network architecture and cross-link dynamics, we simulated a uniaxial stress test, holding the applied stress constant, while varying filament rest length *L*, density *l*_*c*_, elastic modulus *μ*_*e*_ and cross link drag *ξ* (see S1 Table). We measured the elastic modulus, *G*_0_, the effective viscosity, *η*_*c*_, and the timescale *τ*_*c*_ for transition from viscoelastic to effectively viscous behavior, and compared these to theoretical predictions. We observed a transition from viscoelastic to effectively viscous deformation for the entire range of parameter values that we sampled. Our estimate of *G*_0_ from simulation agreed well with the closed form solution *G*_0_ ∼ *μ*/*l*_*c*_ predicted by a previous theoretical model [66] for networks of semi-flexible filaments with irreversible cross-links (Fig 3B).

A simple theoretical analysis of filament networks with frictional cross link slip, operating in the intermediate viscous regime (see S1 Appendix A.2), predicted that the effective viscosity *η*_*c*_ should be proportional to the cross-link drag coefficient and to the square of the number of cross-links per filament:

$$\begin{array}{c} \eta_{c}=4\pi \xi \;{\left(\frac{L}{l_{c}}-1\right)}^{2} \end{array}$$ (8)

As shown in Fig 3B, our simulations agree well with this prediction for a large range of sampled network parameters. Finally, for many linear viscoelastic materials, the ratio of effective viscosity to the elastic modulus *η*_*c*_/*G*_0_ sets the timescale for transition from elastic to viscous behavior [67]. Combining our approximations for *G*_0_ and *η*_*c*_, we predict a transition time, *τ*_*c*_ ≈ *L*^2^*ξ*/*l*_*c*_*μ*. Measuring the time at which the strain rate became nearly constant (i.e. *γ* ∼ *t*^*n*^ with *n* > 0.8) yields an estimate of *τ*_*c*_ that agrees well with this prediction over the entire range of sampled parameters (Fig 3C). Thus the passive response of filament networks with frictional cross link drag is well-described on short (viscoelastic) to intermediate (viscous) timescales by an elastic modulus *G*_0_, an effective viscosity *η*_*c*_, and a transition timescale *τ*_*c*_, with well-defined dependencies on network parameters. On longer timescales, the cumulative decrease in filament density leads to material failure.

#### Filament turnover allows sustained large-scale viscous flow and defines two distinct flow regimes

To characterize how filament turnover shapes the passive network response to an applied force, we introduced a simple form of turnover in which entire filaments disappear at a rate *k*_*diss*_*ρ*, where *ρ* is the filament density, and new unstrained filaments appear with a fixed rate per unit area, *k*_*ass*_. Absent deformation, filament density will equilibrate to a steady state value, *ρ*_0_ = *k*_*ass*_/*k*_*diss*_, with time constant *τ*_*r*_ = 1/*k*_*diss*_. However, in networks deforming under extensional stress, density will be shaped by both turnover and network extension, and changes in density will feed back on deformation through the density-dependence of effective viscosity (Fig 3B).

**Fig 4:**
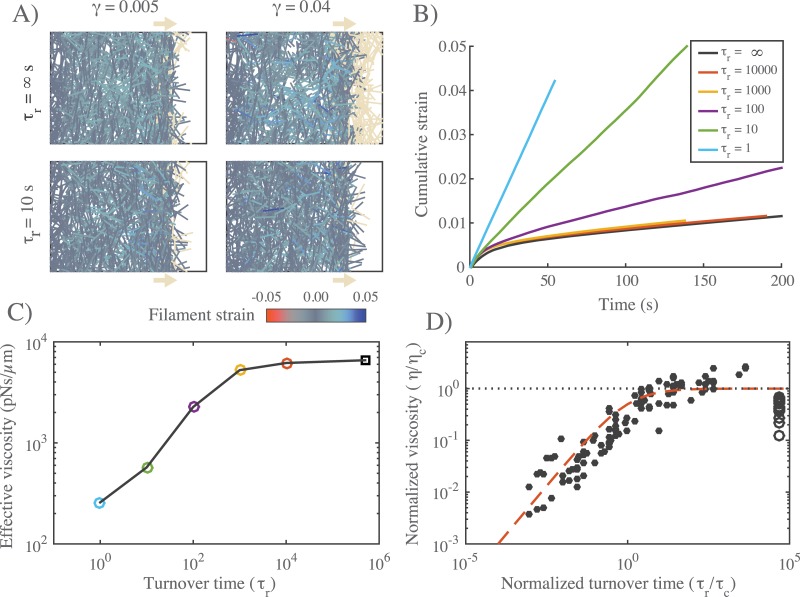
Filament turnover defines two regimes of effectively viscous flow. **A)** Comparison of 12 × 20*μm* networks under 0.1 *pN*/*μm* extensional stress without (top) and with (bottom) filament turnover. Both images are taken when the networks had reached a net strain of 0.04. For clarity, filaments that leave the domain of applied stress are greyed out. **B)** Plots of strain vs time for identical networks with different rates of filament turnover. Network parameters: *L* = 5 *μm*, *l*_*c*_ = 0.5 *μm*, *ξ* = 1 *nN* ⋅ *s*/*μm*. **C)** Plot of effective viscosity vs turnover time derived from the simulations shown in (B). Square dot is the *τ*_*r*_ = ∞ condition. **D)** Plot of normalized effective viscosity (*η*/*η*_*c*_) vs normalized turnover time (*τ*_*r*_/*τ*_*c*_) for a large range of network parameters and turnover times. For *τ*_*r*_ ≪ *τ*_*c*_, the viscosity of the network becomes dependent on turnover time. Red dashed line indicates the approximation given in eq 9 for *m* = 3/4.

To examine the consequences of these effects, we simulated a uniaxial stress test for different values of *τ*_*r*_, while holding *ρ*_0_ and all other parameters fixed (Fig 4A–4C). For large *τ*_*r*_, network extension was accompanied by a continuous decrease in filament density, leading ultimately to loss of connectivity and material failure (S2 Fig). For lower values of *τ*_*r*_, the network approached a steady state characterized by continuous extension at a constant density and strain rate (Fig 4B, S2 Fig). Analysis of a simple coarse-grained model (S1 Appendix A.3) revealed how this behavior emerges from a competition between density equilibration via turnover and the decrease in density, and thus effective viscosity, during extension. For *τ*_*r*_ greater than a critical lifetime *τ*_*crit*_, the latter effect dominates, leading to a runaway decrease in filament density, loss of connectivity and material failure. For *τ*_*r*_ < *τ*_*crit*_, the coarse-grained model predicts the existence of a dynamically stable steady state characterized by continuous extension at constant density and strain rate (S1 Appendix A.3), as observed in our simulations (S3 Fig).

For *τ*_*r*_ < *τ*_*crit*_, we observed two distinct steady state flow regimes (Fig 4B and 4C). For intermediate values of *τ*_*r*_, effective viscosity remains constant with decreasing *τ*_*r*_. However, below a certain value of *τ*_*r*_ (≈ 10^3^ for the parameters used in Fig 4C), effective viscosity decreased monotonically with further decreases in *τ*_*r*_. To understand what sets the timescale for transition between these two regimes, we measured effective viscosity at steady steady for a wide range of network parameters (*L*, *μ*, *l*_*c*_), crosslink drags (*ξ*) and filament turnover times (Fig 4D). Strikingly, when we plotted the normalized effective viscosity *η*_*r*_/*η*_*c*_ vs a normalized turnover time *τ*_*r*_/*τ*_*c*_ for all parameter values, the data collapsed onto a single curve, with a transition at *τ*_*r*_ ≈ *τ*_*c*_ between an intermediate turnover regime in which effective viscosity is independent of *τ*_*r*_ and an high turnover regime in which effective viscosity falls monotonically with decreasing *τ*_*r*_/*τ*_*c*_ (Fig 4D).

This biphasic dependence of effective viscosity on filament turnover can be understood intuitively as follows: As new filaments are born, they become progressively stressed as they stretch and reorient under local influence of surrounding filaments, eventually reaching an elastic limit where their contribution to resisting network deformation is determined by effective crosslink drag. The time to reach this limit is about the same as the time, *τ*_*c*_, for an entire network of initially unstrained filaments to reach an elastic limit during the initial viscoelastic response to uniaxial stress, as shown in Fig 2b. For *τ*_*r*_ < *τ*_*c*_, individual filaments do not have time, on average, to reach the elastic limit before turning over; thus the deformation rate is determined by the elastic resistance of partially strained filaments, which increases with lifetime up to *τ*_*r*_ = *τ*_*c*_. For *τ*_*r*_ > *τ*_*c*_, the deformation rate is largely determined by cross-link resistance to sliding of maximally strained filaments, and the effective viscosity is insensitive to further increase in *τ*_*r*_.

These results complement and extend a previous computational study of irreversibly cross-linked networks of treadmilling filaments deforming under extensional stress [68]. Kim et al. identified two regimes of effectively viscous deformation: a “stress-dependent” regime in which filaments turnover before they become strained to an elastic limit and deformation rate is proportional to both applied stress and turnover rate; and a “stress-independent” regime in which filaments reach an elastic limit before turning over and deformation rate depends only on the turnover rate. The fast and intermediate turnover regimes that we observe here correspond to the stress-dependent and independent regimes described by Kim et al., but with a key difference. Without filament turnover, Kim et al.’s model predicts that a network cannot deform beyond its elastic limit. In contrast, our model predicts viscous flow at low turnover, governed by an effective viscosity that is set by cross-link density and effective drag. Thus our model provides a self-consistent framework for understanding how crosslink unbinding and filament turnover contribute separately to viscous flow and connects these contributions directly to previous theoretical descriptions of cross-linked networks of semi-flexible filaments.

In summary, our simulations predict that filament turnover allows networks to undergo viscous deformation indefinitely, without loss of connectivity or material failure, over a wide range of different effective viscosities and deformation rates. For *τ*_*r*_ < *τ*_*crit*_, the observed dependence of effective viscosity on filament lifetime can be represented phenomenologically by a simple equation of the form:

$$\begin{array}{c} \eta =\frac{\eta_{c}}{1+{\left(\tau_{c}/\tau_{r}\right)}^{m}} \end{array}$$ (9)

where the exponent *m* = 3/4 was chosen to yield a good fit to data in (Fig 4D). For *τ*_*r*_ ≫ *τ*_*c*_, *η* ≈ *η*_*c*_: effective viscosity depends on crosslink density and effective crosslink drag, independent of changes in turnover rate. For *τ*_*r*_ ≪ *τ*_*c*_, effective viscosity is governed by the level of elastic stress on network filaments, and becomes strongly dependent on filament lifetime: *η* ∼ *η*_*c*_(*τ*_*r*_/*τ*_*c*_)^*m*^. The origins of the *m* = 3/4 scaling remain unclear (see Discussion).

### Filament turnover allows persistent stress buildup in active networks

#### Asymmetric filament compliance and spatial heterogeneity in motor acitvity is sufficient for macroscopic contraction

**Fig 5:**
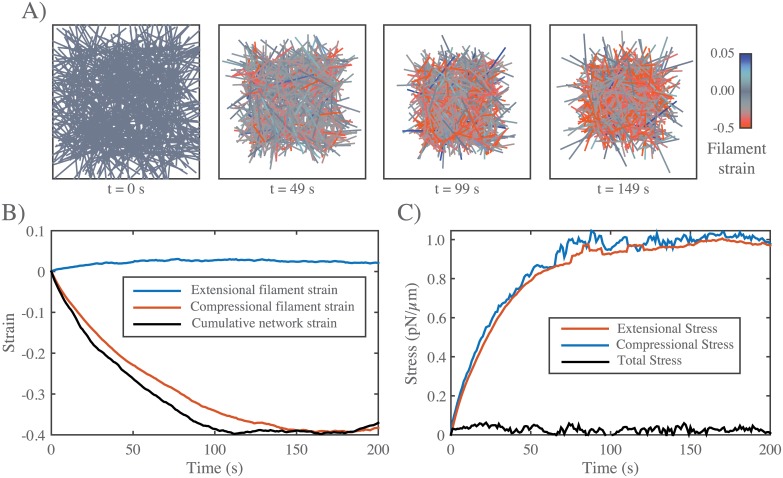
In the absence of filament turnover, active networks with free boundaries contract and then stall. **A)** Simulation of an active network with free boundaries. Colors represent strain on individual filaments as in previous figures. Note the buildup of compressive strain as contraction approaches stall between 100 s and 150 s. Network parameters: *L* = 5 *μm*, *l*_*c*_ = 0.3 *μm*, *ξ* = 100 *pN* ⋅ *s*/*μm*, *υ* = 10 *pN*. **B)** Plots showing time evolution of total network strain (black) and the average extensional (blue) or compressional (red) strain on individual filaments. **C)** Plots showing time evolution of total (black) extensional (blue) or compressional (red) stress. Note that extensional and compressional stress remain balanced as compressional resistance builds during network contraction.

Previous work [37, 39, 69] identifies asymmetric filament compliance and spatial heterogeneity in motor activity as minimal requirements for macroscopic contraction of disordered networks. To confirm that our simple implementation of these two requirements (see Models section) is sufficient for macroscopic contraction, we simulated active networks that are unconstrained by external attachments, varying filament length, density, crosslink drag and motor activity. We observed qualitatively similar results for all choices of parameter values: Turning on motor activity in an initially unstrained network induced rapid initial contraction, followed by a slower buildup of compressive stress (and strain) on individual filaments, and an ∼exponential approach to stall (Fig 5). On longer timescales, polarity sorting of individual filaments, as previously described [41, 43–45] accompanied network expansion (see S2 Video).

During the rapid initial contraction, the increase in network strain closely matched the increase in mean compressive strain on individual filaments Fig 5B, as predicted theoretically [37, 38] and observed experimentally [39]. Contraction required asymmetric filament compliance and spatial heterogeneity of motor activity (*μ*_*e*_/*μ*_*c*_ > 1, *ϕ* < 1, S3A Fig). Thus our model captures a minimal mechanism for bulk contractility in disordered networks through asymmetric filament compliance and dispersion of motor activity.

#### Active networks cannot sustain stress against a fixed boundary in the absence of filament turnover

**Fig 6:**
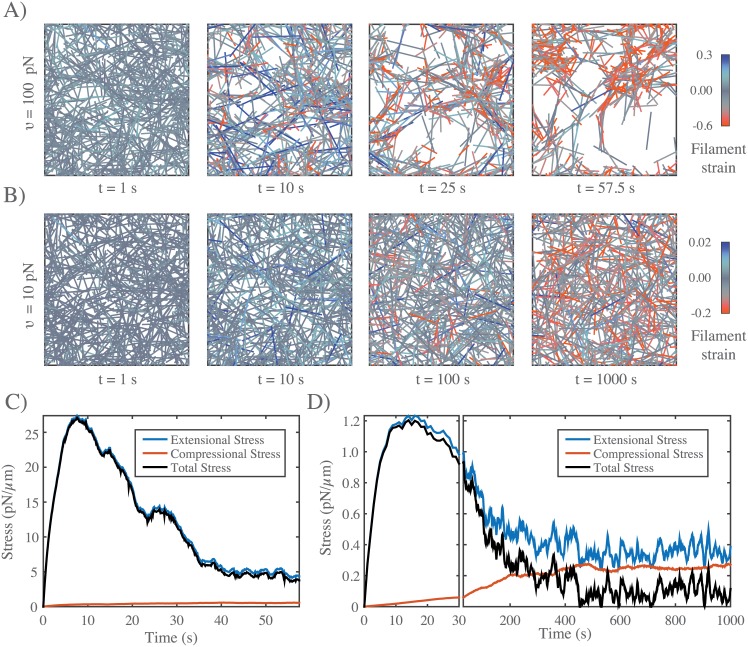
In the absence of filament turnover, active networks cannot sustain continuous stress against a fixed boundary. **A)** Simulation of an active network with fixed boundaries. Rearrangement of network filaments by motor activity leads to rapid network fragmentation. Network parameters: *L* = 5 *μm*, *l*_*c*_ = 0.3 *μm*, *ξ* = 100 *pN* ⋅ *s*/*μm*, *υ* = 100 *pN*. **B)** Simulation of the same network, with the same parameter values, except with ten-fold lower motor activity *υ* = 10 *pN*. In this case, the distribution of filaments remains more uniform, and network connectivity is maintained in the sense that each filament maintains overlap with many others. Note the progressive shift towards less extensional and greater compressional strain on individual filaments (see color map). **C)** Plots of total network stress and the average extensional (blue) and compressional (red) stress vs. time on individual filaments for the simulation shown in (A). Rapid buildup of extensional stress allows the network transiently to exert force on its boundary, but this force decays and this decay is closely associated with a decrease in extensional stress, reflecting the breakdown in network connectivity. **D)** Plots of total network stress and the average extensional (blue) and compressional (red) stress vs. time on individual filaments for the simulation shown in (b). Rapid buildup of extensional stress allows the network transiently to exert force on its boundary. However, stress decays at longer times as decreasing extensional stress and increasing compressional stress approach balance. Note the different time scales used for plots and subplots in **C)** and **D)** to emphasize the similar timescales for force buildup, but different timescales for force dissipation.

During cortical flow, regions with high motor activity contract against passive resistance from neighboring regions with lower motor activity. To understand how the active stresses that drive cortical flow are shaped by external resistance, we analyzed the buildup and maintenance of contractile stress in active networks contracting against a rigid boundary. We simulated active networks contracting from an initially unstressed state against a fixed boundary (Fig 6A and 6B), and monitored the time evolution of mean extensional (blue), compressional (red) and total (black) stress on network filaments (Fig 6C and 6D). We focused initially on the scenario in which there is no, or very slow, filament turnover, sampling a range of parameter values controlling filament length and density, motor activity, and crosslink drag.

For all parameters, total stress built rapidly to a peak value *σ*_*m*_, and then decayed over time (Fig 6C and 6D). The rapid initial increase was determined largely by a rapid buildup of extensional stress (Fig 6C and 6D) on a subset of network filaments (Fig 6A and 6B *t* = 10*s*). The subsequent decay involved two forms of local remodeling: for some parameter values, e.g. for higher motor activity (e.g. Fig 6A and 6C), active forces drove rapid network tearing and fragmentation, as previously described [40, 47]. The decay in total stress was closely associated with loss of extensional stress, while buildup of compressive stress made a minor contribution. For other parameter values, (e.g. for lower motor activity as in Fig 6B and 6D), the distribution of filaments remained more uniform, and the decay in total stress was accompanied by a slow decrease in extensional stress, and a slow increase in compressional stress, such that at long times, a low level of residual total stress was sustained by a balance of larger extensional and compressive stresses (see Fig 6D).

Combining dimensional analysis with trial and error, we were able to find empirical scaling relationships describing the dependence of maximum stress *σ*_*m*_ and the time to reach maximum stress *τ*_*m*_ on network parameters and effective crosslink drag ($\sigma_{m}∼\sqrt{\mu_{e}\upsilon }/l_{c}$, $\tau_{m}∼L\xi /\sqrt{\mu_{e}\upsilon }$, S3C and S3D Fig). Although these relationships should be taken with a grain of salt, they are consistent with our simple intuition that the peak stress should increase with motor force (*υ*), extensional modulus (*μ*_*e*_) and filament density (1/*l*_*c*_), and the time to reach peak stress should increase with crosslink drag (*ξ*) and decrease with motor force (*υ*) and extensional modulus (*μ*_*e*_). We were unable to find simple scaling relationships for stress decay, likely because this involves multiple forms of filament rearrangement with different scaling dependencies.

#### Filament turnover allows active networks to exert sustained stress on a fixed boundary

**Fig 7:**
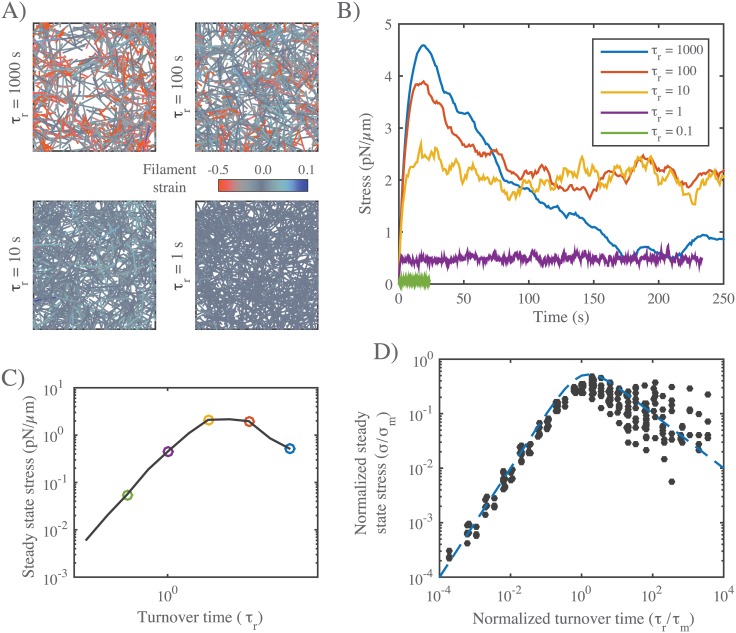
Filament turnover allows active networks to exert sustained stress on a fixed boundary. **A)** Snapshots from simulations of active networks with fixed boundaries and different rates of filament turnover. All other parameter values are the same as in Fig 6A. Note the significant buildup of compressional strain and significant remodeling for longer, but not shorter, turnover times. **B)** Plots of net stress exerted by the network on its boundaries for different recycling times; for long-lived filaments, stress is built rapidly, but then dissipates. Decreasing filament lifetimes reduces stress dissipation by replacing compressed with uncompressed filaments, allowing higher levels of steady state stress; for very short lifetimes, stress is reduced, because individual filaments do not have time to build stress before turning over. **C)** Plots of steady state stress estimated from the simulations in **B)** vs. turnover time. **D)** Plot of normalized steady state stress vs. normalized turnover time for a wide range of network parameters and turnover times. Steady state stress is normalized by the predicted maximum stress *σ*_*m*_ achieved in the absence of filament turnover. Turnover time is normalized by the predicted time to achieve maximum stress *τ*_*m*_, in the absence of filament turnover. Predictions for *σ*_*m*_ and *τ*_*m*_ were obtained from the phenomenological scaling relations shown in (S3B and S3C Fig). Dashed blue line indicates the approximation given in eq 10 for *n* = 1.

Regardless of exactly how active stress is dissipated over time, these results reveal a fundamental limit on the ability of active networks to sustain force against an external resistance in the absence of filament turnover. To understand how this limit can be overcome by filament turnover, we simulated networks contracting against a fixed boundary from an initially unstressed state, for increasing rates of filament turnover (decreasing *τ*_*r*_), while holding all other parameter values fixed (Fig 7A–7C). While the peak stress decreased monotonically with decreasing *τ*_*r*_, the steady state stress showed a biphasic response, increasing initially with decreasing *τ*_*r*_, and then falling off as *τ*_*r*_ → 0. We observed a biphasic response regardless of how stress decays in the absence of turnover, i.e. whether decay involves loss of network connectivity, or local remodeling without loss of connectivity, or both (S4 Fig). Significantly, when we plot normalized steady state stress (*σ*/*σ*_*m*_) vs normalized turnover time (*τ*_*r*_/*τ*_*m*_) for a wide range of network parameters, the data collapse onto a single biphasic response curve, with a peak near *τ*_*r*_/*τ*_*m*_ = 1 (Fig 7D). In particular, for *τ*_*r*_ < *τ*_*m*_, the scaled data collapsed tightly onto a single curve representing a linear increase in steady state stress with increasing *τ*_*r*_. For *τ*_*r*_ > *τ*_*m*_, the scaling was less consistent, although the trend towards a monotonic decrease with increasing *τ*_*r*_ was clear.

As for the passive response this biphasic dependence could be described phenomenologically with a simple equation of the form:

$$\begin{array}{c} \sigma_{ss}=\frac{\sigma_{m}}{{\left(\tau_{r}/\tau_{m}\right)}^{n}+\tau_{m}/\tau_{r}} \end{array}$$ (10)

where the exponent *n* = 1 was chosen to yield a reasonable fit to the data in (Fig 7D) for *τ*_*r*_ > *τ*_*m*_.

These results reveal that filament turnover can “rescue” the dissipation of active stress during isometric contraction due to network remodeling, and they show that, for a given choice of network parameters, there is an optimal choice of filament lifetime that maximizes steady state stress.

We can understand the biphasic dependence of steady state stress on filament lifetime using the same reasoning applied to the case of passive flow: During steady state contraction, the average filament should build and dissipate active stress on approximately the same schedule as an entire network contracting from an initially unstressed state (Fig 7B). Therefore for *τ*_*r*_ < *τ*_*m*_, increasing lifetime should increase the mean stress contributed by each filament. For *τ*_*r*_ > *τ*_*m*_, further increases in lifetime should begin to reduce the mean stress contribution. Directly comparing the time-dependent buildup and dissipation of stress in the absence of turnover, with the dependence of steady state stress on *τ*_*r*_, supports this interpretation (S5 Fig).

### Filament turnover tunes the balance between active stress buildup and viscous stress relaxation to generate flows

**Fig 8:**
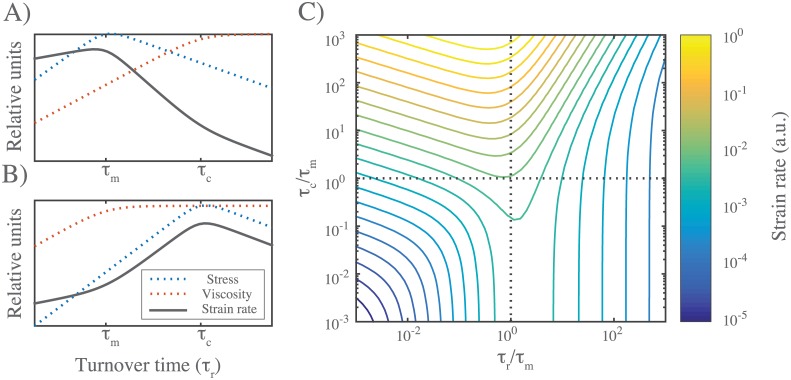
Filament turnover tunes the magnitudes of both effective viscosity and steady state stress. **A)** Dependence of steady state stress, effective viscosity, and resulting strain rate on turnover time *τ*_*r*_ under the condition *τ*_*m*_ < *τ*_*c*_. **B)** Same as (A) but for *τ*_*c*_ < *τ*_*m*_. **C)** Phase diagram for flow rate dependence relative to the two relaxation timescales, *τ*_*r*_ and *τ*_*c*_ normalized by the stress buildup timescale, *τ*_*m*_.

Thus far, we have considered independently how network remodeling controls the passive response to an external stress, or the steady state stress produced by active contraction against an external resistance. We now consider how these two forms of dependence can combine to shape steady state flow produced by spatial gradients of motor activity. To this end, we model a simple scenario in which motor activity is continuously patterned such that the right half network has uniformly high levels of motor activity (controlled by *υ*, with *ϕ* = 0.5), while the left half network has none (*ϕ* = 0). For simplicity, we imposed periodic boundary conditions at left and right boundaries. Under these conditions, with filament turnover, we expect the right half network to contract continuously against a passive resistance from the left half network. Given the highly asymmetric filament compliance, the internal resistance of the right half network to active compression should be negligible compared to the external resistance of the left half network to extension. Thus the steady state flow should be well-described by:

$$\begin{array}{c} \dot{\gamma }=\frac{\sigma_{ss}}{\eta } \end{array}$$ (11)

where *σ*_*ss*_ is the active stress generated by the right half-network (less the internal resistance to filament compression), *η* is the effective viscosity of the left half network and strain rate $\dot{\gamma }$ is measured in the left half-network. Note that strain rate can be related to the steady state flow velocity *v* at the boundary between right and left halves through $v=\dot{\gamma }D_{x}$. Therefore, we can understand the dependence of flow speed on filament turnover and other parameters using the approximate relationships summarized by eqs 9 and 10 for *η* and *σ*_*ss*_. As shown in Fig 8, there are two qualitatively distinct possibilities for the dependence of strain rate on *τ*_*r*_, depending on the relative magnitudes of *τ*_*m*_ and *τ*_*c*_. In both cases, for fast enough turnover (*τ*_*r*_ < min(*τ*_*m*_, *τ*_*c*_)), we expect weak dependence of strain rate on *τ*_*r*_ ($\dot{\gamma }∼\tau_{r}^{1/4}$). For all parameter values that we sampled in this study (which were chosen to lie in a physiological range), *τ*_*m*_ > *τ*_*c*_. Therefore we predict the dependence of steady state strain rate on *τ*_*r*_ shown in Fig 8A.

**Fig 9:**
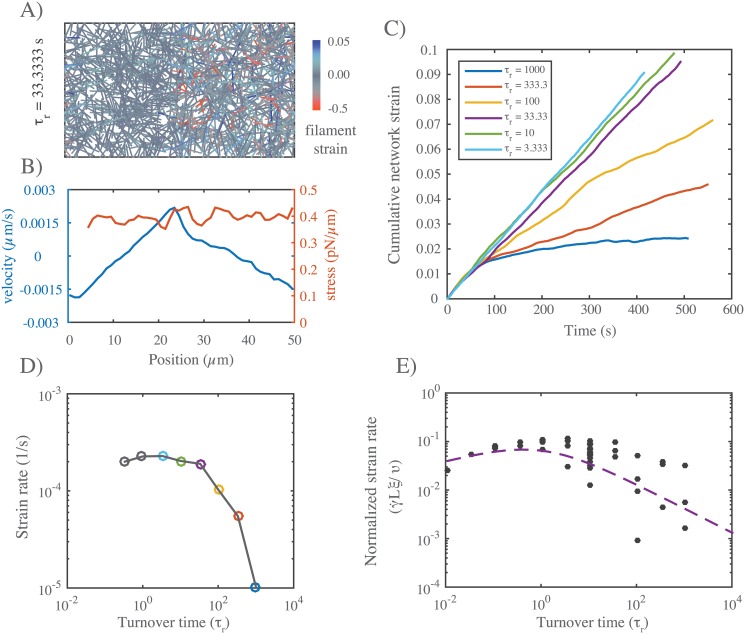
Filament turnover allows sustained flows in response to gradients of motor activity. **A)** Snapshot from a simulation of an asymmetrically contracting network, with motor activity restricted to right half domain, and *τ*_*r*_ = 33. Network parameters are same as in Figs 6 and 7, and *τ*_*m*_ < *τ*_*c*_. **B)** Plots of stress and flow velocity vs position for an analogous simulation (with *τ*_*r*_ = 10) at long times, under ∼ steady state flow. Blue indicates average filament velocity while red represents average network stress, measured as described in the main text. **C)** Graph of cumulative network strain (on the left half-network) vs. time for identical networks with varying turnover rates. **D)** Steady state strain rate on the left half-network as a function of *τ*_*r*_, with colored points corresponding to the data from (C). **E)** Normalized steady state strain rate on the left half network as a function of turnover time *τ*_*r*_. Dashed line is form of dependence predicted by the theoretical arguments shown in Fig 8. Importantly, for all parameter values, *τ*_*m*_ < *τ*_*c*_, corresponding to the scenario in Fig 8A.

To test this prediction, we simulated the simple scenario described above for different values of *τ*_*r*_, with all other parameter values initially fixed at their reference values (Fig 9A). As expected, for all values of *τ*_*r*_, the asymmetric pattern of active contraction gave rise to steady state flow, characterized by continuous contraction of the right half-network and expansion of the left half-network, with peak velocity at the boundaries between right (contracting) and left (expanding) domains (Fig 9B). At long times, the average strain on individual filaments reached a plateau (see S6 Fig), but the cumulative network strain increased linearly with time (Fig 9C), indicating steady state flow with a constant strain rate and peak velocity. Plotting steady state strain rate vs filament lifetime *τ*_*r*_ confirmed the predicted dependence: Steady state strain rates approached zero with increasing *τ*_*r*_; however, for decreasing *τ*_*r*_, steady state strain rates increased steadily, before reaching an approximate plateau on which strain rate varied by less than 15% over more than two decades of variation in *τ*_*r*_ (Fig 9D).

We repeated these simulations for a wider range of parameter values, and saw the same qualitative dependence of $\dot{\gamma }$ on *τ*_*r*_ in all cases. Using eq 9 with *τ*_*r*_ < *τ*_*c*_ and eq 10 with *τ*_*r*_ < *τ*_*m*_, and the theoretical or empirical scaling relationships found above for *η*_*c*_, *τ*_*c*_, *σ*_*m*_ and *τ*_*m*_, we predict a simple scaling relationship for $\dot{\gamma }$ (for small *τ*_*r*_, see Fig 9D):

$$\begin{array}{c} \dot{\gamma }=\frac{\upsilon }{\xi L}{\left(\tau_{r}\right)}^{1/4} \end{array}$$ (12)

Indeed, when we plot the steady state measurements of $\dot{\gamma }$, normalized by *υ*/*ξL*, for all parameter values, the data collapse onto a single curve for small *τ*_*r*_. Thus. our simulations identify a flow regime, characterized by sufficiently fast filament turnover, in which the steady state flow speed is buffered against variation in turnover, and has a relatively simple dependence on other network parameters.

## Discussion

Cortical flows arise through a dynamic interplay of force production and dissipation within cross-linked actomyosin networks. Here we combined computer simulations with simple theoretical analysis to explore how this interplay depends on motor activity, crosslink dynamics, network architecture and filament turnover. Our results reveal two essential requirements for filament turnover during cortical flow: (a) to allow the continuous relaxation of elastic resistance without catastrophic loss of network connectivity and (b) to prevent the dissipation of active stress through local network rearrangements. We find that biphasic dependencies of active stress and passive relaxation on filament lifetime define multiple modes of steady state flow with distinct dependencies on network parameters and filament turnover.

We identify two distinct modes of passive response to uniaxial stress: a low turnover mode in which filaments strain to an elastic limit before turning over, and effective viscosity depends on crosslink density and effective crosslink friction, and a high turnover mode in which filaments turn over before reaching an elastic limit and effective viscosity is proportional to elastic resistance and approximately proportional to filament lifetime. We note that the weakly sub-linear dependence of effective viscosity on filament lifetime that we observe in the high turnover regime may simply reflect a failure to capture very local modes of filament deformation, since a previous study [68] in which filaments were represented as connected chains of smaller segments predicted linear dependence of effective viscosity on filament lifetime. While previous studies have emphasized individual roles for cross-link unbinding or filament turnover in stress relaxation [7, 13, 22], here we have capture their distinct contributions within a single self-consistent modeling framework.

Our simulations confirm the theoretical prediction [37, 39, 69] that spatial heterogeneity of motor activity and asymmetric filament compliance are sufficient to support macroscopic contraction of unconstrained networks. However, under isometric conditions, and without filament turnover, our simulations predict that active stress cannot be sustained. On short timescales, motor forces drive local buildup of extensional stress, but on longer timescales, active local filament rearrangements lead, invariably, to a decay in active stress. These rearrangements can lead to macroscopic network tearing and fragmentation, as previously described [40, 47]. However, stress decay can also occur when the distribution of network filaments remains more uniform and the network remains globally connected in the sense that every filament overlaps, and can exchange frictional crosslink forces, with many others. Under these conditions, network rearrangements involve a slower rebalancing of extensile and compressive forces on network filaments. Our results suggest that when filaments can slide relative to one another, the motor forces that produce active stress will drive changes either in connectivity, or in the distributions of forces along individual filaments, or both, that inevitably lead to a decrease in active stress. Thus for contractile networks to maintain isometric tension on long timescales, they must either form stable crosslinks to prevent filament rearrangements, or they must continuously turnover network filaments (or active motors) to renew the local potential for production of active stress.

Indeed, our simulations predict that filament turnover is sufficient for maintenance of active stress. As in the passive case, they predict biphasic dependence of steady state stress on filament turnover: For short-lived filaments (*τ*_*r*_ < *τ*_*m*_), steady state stress increases linearly with filament lifetime because filaments have more time to build towards peak extensional stress before turning over. For longer-lived filaments (*τ*_*r*_ > *τ*_*m*_), steady state stress decreases monotonically with filament lifetime because local rearrangements decrease the mean contributions of longer lived filaments. These findings imply that for cortical networks that sustain contractile stress under approximately isometric conditions, tuning filament turnover can control the level of active stress, and there will be an optimal turnover rate that maximizes the stress, all other things equal. This may be important, for example in early development, where contractile forces produced by cortical actomyosin networks maintain, or drive slow changes in, cell shape and tissue geometry [7, 70].

For cortical networks that undergo steady state flows driven by spatial gradients of motor activity, our simulations predict that the biphasic dependencies of steady state stress and effective viscosity on filament lifetime define multiple regimes of steady state flow, characterized by different dependencies on filament turnover (and other network parameters). In particular, the approximately linear dependencies of steady state stress and effective viscosity on filament lifetime for short-lived filaments define a fast turnover regime in which steady state flow speeds are buffered against variations in filament lifetime, and are predicted to depend in a simple way on motor activity and crosslink resistance. Measurements of F-actin turnover times in cells that undergo cortical flow [32, 71–75] suggests that they may indeed operate in this fast turnover regime. For reference values of model parameters, the steady state strain rates predicted for the high turnover regime (≈ 2*x*10^−^4/*sec*) are approximately ten-fold lower than those measured in polarized *C. elegans* zygotes (≈ 1 − 2*x*10^−^3/*sec*) [6, 11, 76]. This is reasonable agreement, given uncertainties about these reference values. For example, our reference value for effective crosslink friction *ξ* is 10-100-fold higher than friction coefficients measured for single crosslinkers and molecular motors *in vitro* [77, 78]. A ten-fold lower value for this parameter would yield a ten-fold increase in the predicted steady state strain rate (12). Interestingly, recent studies in *C. elegans* embryos suggests that cortical flow speeds are surprisingly insensitive to depletion of factors (ADF/Cofilin) that govern filament turnover [11], again consistent with our model’s predictions. Stronger tests of our model’s predictions will require more systematic analyses of how flow speeds vary with filament and crosslink densities, motor activities, and filament lifetimes.

## Supporting information

S1 Appendix Code reference and supplementary methods. **A.1)** Reference to simulation and analysis code. **A.2)** Derivation of effective viscosity. **A.3)** Identification of a critical turnover timescale for steady state flow.
(PDF)

S1 Table Parameter values. List of parameter values used for each set of simulations.
(PDF)

S1 Fig Fast viscoelastic response to extensional stress. Plots of normalized cumulative strain vs time during the elastic phase of deformation in passive networks under extensional stress. Measured strain is normalized by the equilibrium strain predicted for a network of elastic filaments without crosslink slip *γ*_*eq*_ = *σ*/*G*_0_ = *σ*/(2*μ*/*l*_*c*_).
(PDF)

S2 Fig Filament turnover limits density decrease under extensional strain and allows continuous flow without material failure. **A)** Plots of cumulative strain vs time for different turnover times (see inset in (B)). Note the increase in strain rates with decreasing turnover time. **B)** Plots of filament length density vs time for different turnover times *τ*_*r*_. For long to intermediate *τ*_*r*_, simulations predict an approximately linear decrease in length density with time, at a rate that decreases with decreasing *τ*_*r*_, leading ultimately to loss of connectivity and material failure. For lower *τ*_*r*_, length densities approach steady state values at longer times. These results match the predictions of the coarse grained analysis in Appendix section A.3.
(PDF)

S3 FigMechanical properties of active networks.**A)** Free contraction requires asymmetric filament compliance, and total network strain increases with the applied myosin force *υ*. Note that the maximum contraction approaches an asymptotic limit as the stiffness asymmetry approaches a ratio of approximately 100. **B)** Maximum stress achieved during isometric contraction, *σ*_*m*_, scales approximately with $\sqrt{\mu_{e}\upsilon }/l_{c}$. **C)** Time to reach max stress during isometric contraction scales approximately with $L\xi /\sqrt{\mu_{e}\upsilon }$. Scalings for *σ*_*m*_ and *τ*_*m*_ were determined empirically by trial and error, guided by dimensional analysis.(PDF)

S4 Fig Filament turnover prevents loss of connectivity and local tearing of active networks. **A)** An active network undergoing large scale deformations due to active filament rearrangements. **B)** The same network as in (A) but with a shorter filament turnover time. **C)** Plots of internal stress vs time for the network in (A). **D)** Plots of internal stress vs time for the network in (B).
(PDF)

S5 Fig Bimodal dependence on turnover time matches bimodal buildup and dissipation of stress in the absence of turnover. **A)** Bimodal buildup of stress in a network with very slow turnover (*τ*_*r*_ = 1000*s*). **B)** Steady state stress for networks with same parameters as in (A), but for a range of filament turnover times.
(PDF)

S6 Fig Average filament strain plateaus during steady state flow. Plots of average filament strain vs time for the network simulation comparable to those shown in (Fig 9A and 9B). in which motor activity is limited to the right-half domain and filament turnover time is *τ*_*r*_ = 10*s*. Blue curve indicates average strain on all extended filaments; red curve indicates average strain on all compressed filaments; yellow curve indicates average strain on all filaments.
(PDF)

S1 Video Extensional strain in passive networks. Movie of simulation setup shown in Fig 2. Colors are the same as in figure.
(MOV)

S2 Video Active networks contracting with free boundaries. Movie of simulation setup shown in Fig 5. Colors are the same as in figure.
(MOV)
