# Supplementary material for: Filament turnover tunes both force generation and dissipation to control long-range flows in a model actomyosin cortex
Source: PLoS Comput Biol. 2017 Dec 18;13(12):e1005811. doi: 10.1371/journal.pcbi.1005811 (PMC5757993; doi:10.1371/journal.pcbi.1005811)
Supplement: S1 Appendix — A.1) Reference to simulation and analysis code. A.2) Derivation of effective viscosity. A.3) Identification of a critical turnover timescale for steady state flow. (PDF) [file pcbi.1005811.s001.pdf]

S1 Appendix

A.1 Simulation and Analysis Code Available Online

All of the simulation and analysis code for generating the figures in this paper is available online. To find the source code please visit our Github repository at

https://github.com/wmcfadden/activnet

A.2 Steady-state Approximation of Effective Viscosity

We begin with a calculation of a strain rate estimate of the effective viscosity for a network described by our model in the limit of highly rigid filaments. We carry this out by assuming we have applied a constant stress along a transect of the network. With moderate stresses, we assume the network reaches a steady state affine creep. In this situation, we would find that the stress in the network exactly balances the sum of the drag-like forces from cross-link slip. So for any transect of length D, we have a force balance equation.

$$\sigma = \frac{1}{D} \sum_{filaments} \sum_{crosslinks} \xi \cdot (\mathbf{v_i}(\mathbf{x}) - \mathbf{v_j}(\mathbf{x})) \tag{1}$$

where $\mathbf{v_i}(\mathbf{x}) - \mathbf{v_j}(\mathbf{x})$ is the difference between the velocity of a filament, i, and the velocity of the filament, j, to which it is attached at the cross-link location, $\mathbf{x}$. We can convert the sum over cross-links to an integral over the length using the average density of cross-links, $1/l_c$ and invoking the assumption of (linear order) affine strain rate, $\mathbf{v_i}(\mathbf{x}) - \mathbf{v_j}(\mathbf{x}) = \dot{\gamma} x$. This results in

$$\begin{aligned}\sigma = \frac{1}{D} \sum_{filaments} \int_0^L \xi \cdot \ (\mathbf{v_i}(\mathbf{s}) - \mathbf{v_j}(\mathbf{s}))\, \frac{ds \cos\theta}{l_c} \\ = \sum_{filaments} \frac{\xi \dot{\gamma} L}{l_c} \cos\theta \cdot (x_l + \frac{L}{2}\cos\theta)\end{aligned} \tag{2}$$

Here we have introduced the variables x_l, and θ to describe the leftmost endpoint and the angular orientation of a given filament respectively. Next, to perform the sum over all filaments we convert this to an integral over all orientations and endpoints that intersect our line of stress. We assume for simplicity that filament stretch and filament alignment are negligible in this low strain approximation. Therefore, the max distance for the leftmost endpoint is the length of a filament, L, and the maximum angle as a function of endpoint is $\arccos(x_l/L)$. The linear density of endpoints is the constant D/l_cL so our integrals can be rewritten as this density over x_l and θ between our maximum and minimum allowed bounds.

$$\sigma = \frac{1}{D}\int_0^L dx_l \int_{-\arccos(\frac{x_l}{L})}^{\arccos(\frac{x_l}{L})} \pi d\theta \frac{\xi\dot{\gamma}L}{l_c} \cdot \frac{D}{Ll_c} \cdot (x_l\cos\theta + \frac{L}{2}cos^2\theta) \tag{3}$$

Carrying out the integrals and correcting for dangling filament ends leaves us with a relation between stress and strain rate.

$$\sigma = 4\pi\left(\frac{L}{l_c} - 1\right)^2 \xi\dot{\gamma} \tag{4}$$

We recognize the constant of proportionality between stress and strain rate as a viscosity (in 2 dimensions). Therefore, our approximation for the effective viscosity, η_c, at steady state creep in this low strain limit is

$$\sigma = 4\pi\left(\frac{L}{l_c} - 1\right)^2 \xi \tag{5}$$

A.3 Critical filament lifetime for steady state filament extension

We seek to determine a critical filament lifetime, τ_{crit} , below which the density of filaments approaches a stable steady state under constant extensional strain. To this end, let ρ be the filament density (i.e. number of filaments per unit area). We consider a simple coarse grained model for how ρ changes as a function of filament assembly at rate k_{ass}, filament disassembly at rate k_{diss}, ρ and dilution of filaments by extensional flow at a rate $\dot{\gamma}\rho$. Accordingly, we write:

$$\frac{d\rho}{dt} = k_{ass}\rho_0 - k_{diss}\rho - \dot{\gamma}\rho \tag{6}$$

Using $\rho_0 = \frac{k_{ass}}{k_{diss}}$, $\tau_r = \frac{1}{k_{diss}}$, and $\dot{\gamma} = \frac{\sigma}{\eta_c}$, we rewrite equation 6 as:

$$\frac{d\rho}{dt} = \frac{1}{\tau_r}\left(\rho_0 - \rho - \frac{\sigma\tau_r}{\eta_c(\rho)}\rho\right) \tag{7}$$

where $\eta_c = \eta_c(\rho)$ on the right hand side reflects the dependence of effective viscosity on network density. The strength of this dependence determines whether there exists a stable steady state, representing continuous flow. Using $\eta_c(\rho) \sim \xi\left(\frac{L}{l_c(\rho)} - 1\right)^2$ from above (ignoring the numerical prefactor), and $\rho \sim \frac{2}{Ll_c(\rho)}$, we obtain:

$$\frac{d\rho}{dt} = \frac{1}{\tau_r}\left(\rho_0 - \rho - \frac{\sigma\tau_r}{\xi(\rho L^2/2 - 1)^2}\rho\right) \tag{8}$$

Defining the a normalized density $X = \rho L^2/2$, normalized time $\tau = \frac{t}{\tau_r}$, normalized filament lifetime $A = \frac{\sigma\tau_r}{\xi}$, and parameter $X_0 = \rho_0 L^2/2$, equation 8 becomes

$$\frac{dX}{dt} = X_0 - X - \frac{AX}{(X-1)^2} \tag{9}$$

Fig. A.1 plots the right-hand-side of equation 9 versus X for several values of the normalized filament lifetime, A. For sufficiently large A, there is no stable state, i.e. flow will reduce density until connectivity is lost. However, as A decreases below a critical value, A_{crit}, a stable steady state appears. Note that when $A = A_{crit}$, $\frac{dX}{dt}$ is tangent to the X-axis at X = X*. Accordingly, to determine X*, we solve

$$0 = \frac{d}{dX}\left(X_0 - X - \frac{AX}{(X-1)^2}\right) = 1 - \frac{A}{(X-1)^2} + \frac{2AX}{(X-1)^3} \tag{10}$$

Equation 10 can be rewritten as cubic polynomial

$$0 = X^3 - 3X^2 + (3 - A)X - (A - 1) \tag{11}$$

which has a single positive real root

$$X^* = 1 + A^{1/3}\left[\left(1 + \sqrt{1 - A/27}\right)^{1/3} + \left(1 - \sqrt{1 - A/27}\right)^{1/3}\right] \tag{12}$$

Note that a purely real X* exists for all $A > 0$, even for $A > 27$, because the imaginary components from the two radicals cancel exactly.

In principle, A_{crit} is determined by setting equation 12 equal to zero, setting X =

X*, and solving for A. The form of equation 12 makes this challenging to do analytically. However, it is straight-forward to determine A_{crit} computationally from equations 9 and 12, for any $X_0 > 0$, for example by plotting the right-hand-side of equation 9 as a function of A, with X determined by equation 12, and looking for the intersection of the curve with the A-axis.

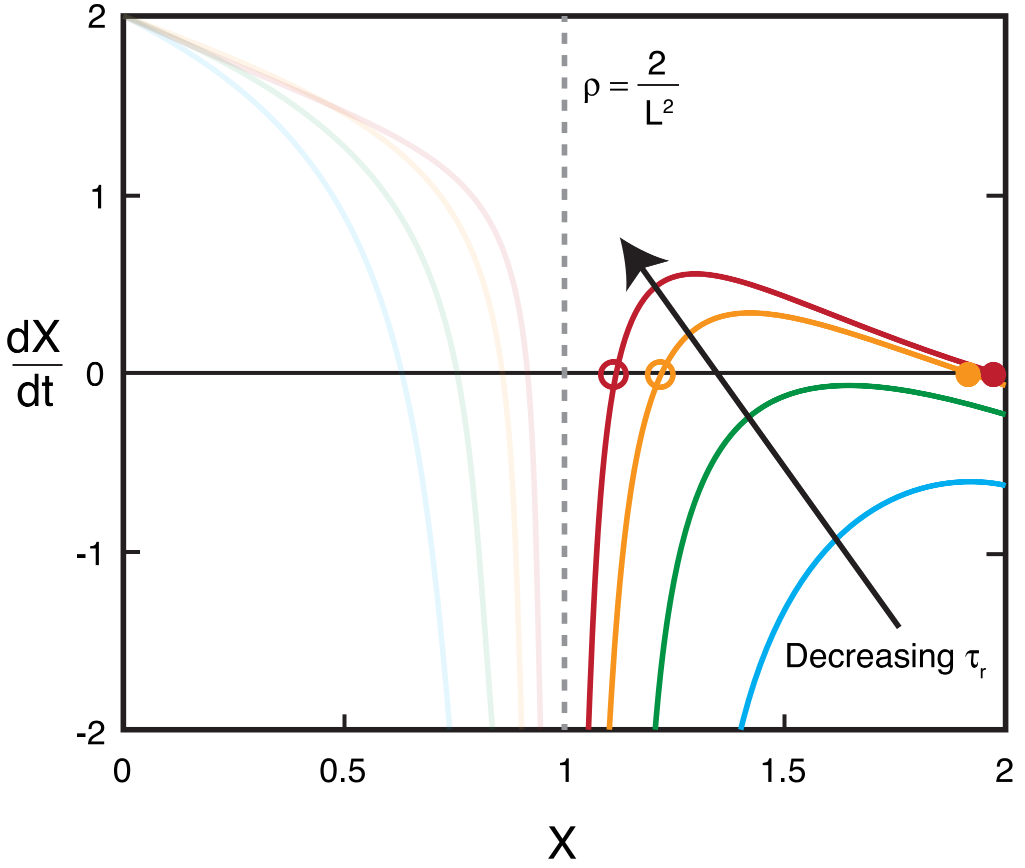

Fig. A.1 Graphical analysis of the time evolution of network density. Phase portraits of the first order ODE describing the time evolution of the normalized density, $\frac{dX}{dt} = X_0 - X - \frac{AX}{(X-1)^2}$, where $X = \rho L^2/2$, $\tau = \frac{t}{\tau_r}$, $A = \frac{\sigma \tau_r}{\xi}$, and $X_0 = \rho_0 L^2/2$. Individual curves represent different values of τ_r. Cool colors correspond to large τ_r, and warm colors correspond to small τ_r. Fixed points corresponding to steady-state densities are represented by zero crossings $\left(\frac{dX}{dt} = 0\right)$. Open and closed circles represent unstable and stable steady states, respectively. The dashed line at $X = 1$ corresponds to a filament density at which the average number of filament overlaps is one. Below this density, the network cannot transmit stress or support coherent flow. Thus we ignore fixed points in the region $X < 1$ (curves showing $\frac{dX}{dt}$ for $X < 1$ are dimmed out). For $\tau_r < \tau_{crit}$, there are two crossings (orange, red): the rightmost crossing represents a stable steady state.
