# Supplementary material for: Filament turnover tunes both force generation and dissipation to control long-range flows in a model actomyosin cortex
Source: PLoS Comput Biol. 2017 Dec 18;13(12):e1005811. doi: 10.1371/journal.pcbi.1005811 (PMC5757993; doi:10.1371/journal.pcbi.1005811)
Supplement: S1 Table — List of parameter values used for each set of simulations. (PDF) [file pcbi.1005811.s002.pdf]

S1 Table.

Table 1. Parameter values sampled for individual figures

Parameter	Units	**Figure 3**	**Figure 4**	**Figure S3a,b**	**Figure S3c,d**	**Figure 7**	**Figure 9**
L	μm	$1, 3, 5, 7, 10$	3	$3, 5$	$3, 5$	5	$3, 5, 8$
l_c	μm	$0.2, 0.3, 0.5, 0.8$	$0.3, 0.5$	0.3	$0.15, 0.2, 0.3, 0.4$	$0.2, 0.3$	$0.15, 0.2, 0.3, 0.4$
μ_e/μ_c		100	100	$3 - 300$	100	100	100
μ_e	pN	100	100	100	$10 - 300$	100	100
μ_c	pN	1	1	$1 - 30$	1	1	1
ξ	$\frac{pNs}{\mu m}$	$10 - 100$	$5, 10, 100$	$1, 10, 100$	$10, 100$	$10, 100, 330$	$10 - 100$
υ	pN			$10, 30, 100$	$10, 100$	$10, 100, 300$	10
ϕ				0.25	0.5	$0.25, 0.75$	0.25
τ_r	s		$0.1 - 10^4$			$0.01 - 10^3$	$0.01 - 10^3$
σ	$\frac{pNs}{\mu m^2}$	$0.02 - 1$	$0.003 - 0.5$				
