# Supplementary figures and images for: Filament turnover tunes both force generation and dissipation to control long-range flows in a model actomyosin cortex
Source: PLoS Comput Biol. 2017 Dec 18;13(12):e1005811. doi: 10.1371/journal.pcbi.1005811 (PMC5757993; doi:10.1371/journal.pcbi.1005811)

Normalized strain  
 $(\gamma / \sigma \cdot 2 \mu / \Lambda_c)$

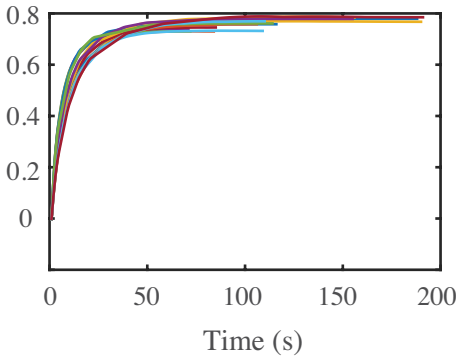

Supplement: S1 Fig — Plots of normalized cumulative strain vs time during the elastic phase of deformation in passive networks under extensional stress. Measured strain is normalized by the equilibrium strain predicted for a network of elastic filaments without crosslink slip γeq = σ/G0 = σ/(2μ/lc). (PDF) [file pcbi.1005811.s003.pdf]

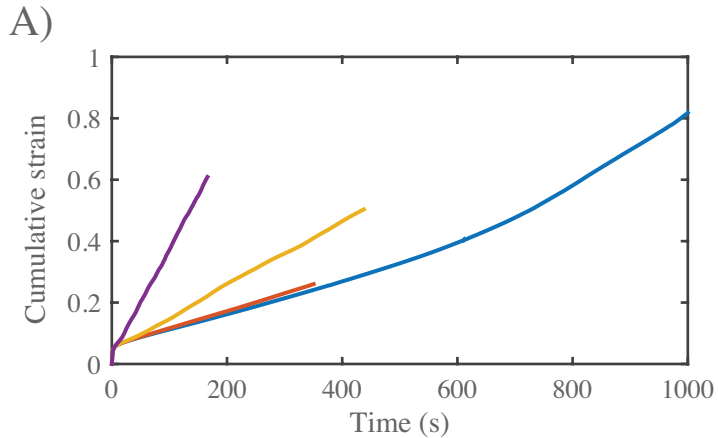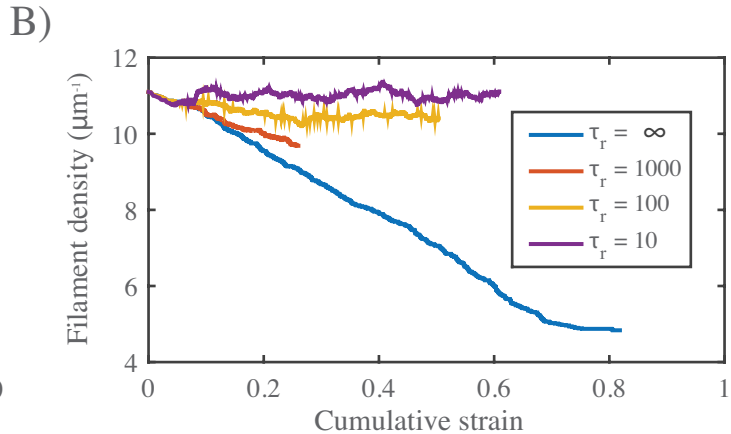

Supplement: S2 Fig — A) Plots of cumulative strain vs time for different turnover times (see inset in (B)). Note the increase in strain rates with decreasing turnover time. B) Plots of filament length density vs time for different turnover times τr. For long to intermediate τr, simulations predict an approximately linear decrease in length density with time, at a rate that decreases with decreasing τr, leading ultimately to loss of connectivity and material failure. For lower τr, length densities approach steady state values at longer times. These results match the predictions of the coarse grained analysis in Appendix section A.3. (PDF) [file pcbi.1005811.s004.pdf]

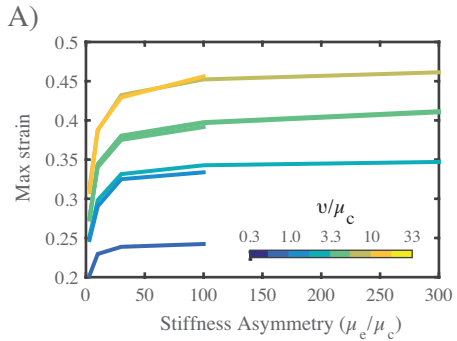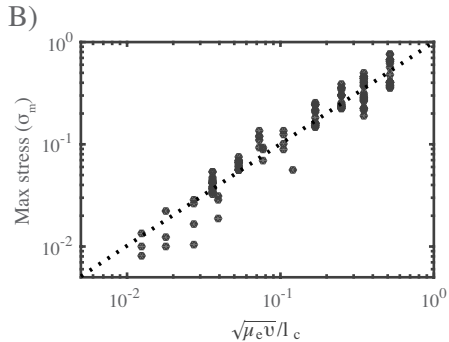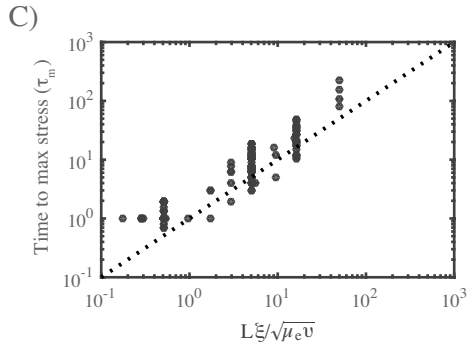

Supplement: S3 Fig — A) Free contraction requires asymmetric filament compliance, and total network strain increases with the applied myosin force υ. Note that the maximum contraction approaches an asymptotic limit as the stiffness asymmetry approaches a ratio of approximately 100. B) Maximum stress achieved during isometric contraction, σm, scales approximately with μeυ/lc. C) Time to reach max stress during isometric contraction scales approximately with Lξ/μeυ. Scalings for σm and τm were determined empirically by trial and error, guided by dimensional analysis. (PDF) [file pcbi.1005811.s005.pdf]

A)

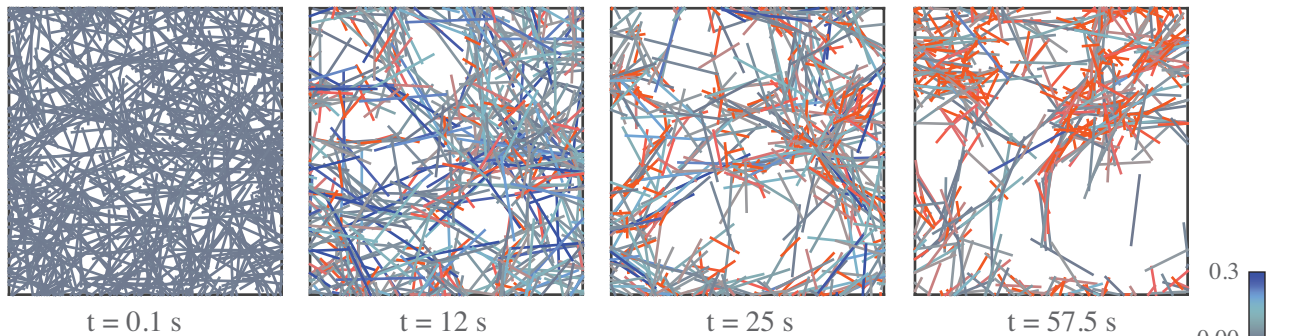

B)

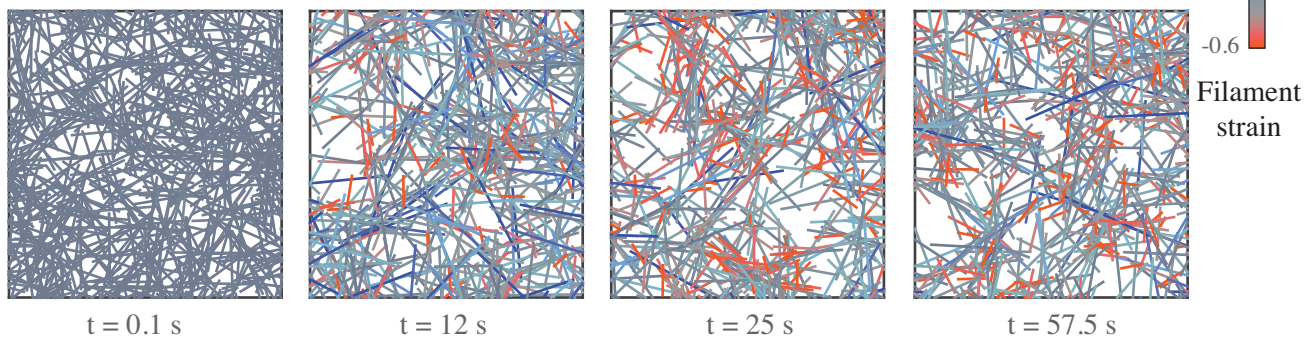

C)

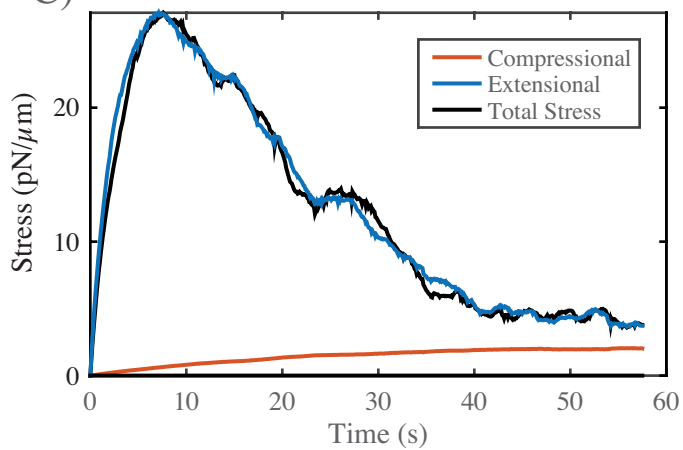

D)

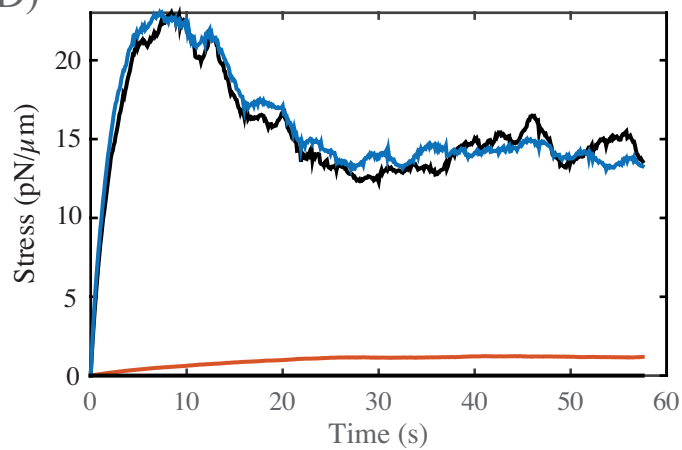

Supplement: S4 Fig — A) An active network undergoing large scale deformations due to active filament rearrangements. B) The same network as in (A) but with a shorter filament turnover time. C) Plots of internal stress vs time for the network in (A). D) Plots of internal stress vs time for the network in (B). (PDF) [file pcbi.1005811.s006.pdf]

A)

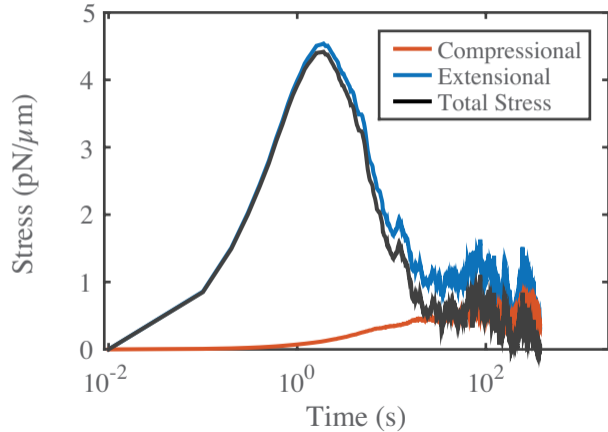

B)

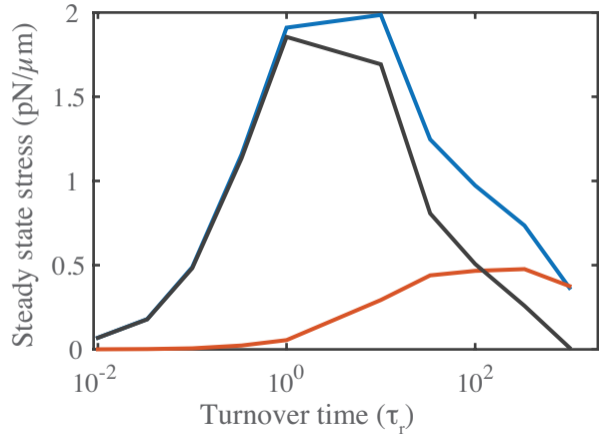

Supplement: S5 Fig — A) Bimodal buildup of stress in a network with very slow turnover (τr = 1000s). B) Steady state stress for networks with same parameters as in (A), but for a range of filament turnover times. (PDF) [file pcbi.1005811.s007.pdf]

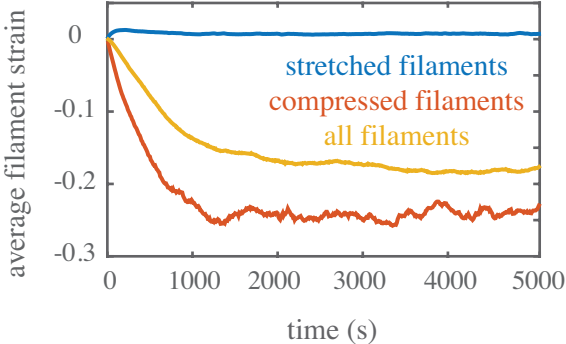

Supplement: S6 Fig — Plots of average filament strain vs time for the network simulation comparable to those shown in (Fig 9A and 9B). in which motor activity is limited to the right-half domain and filament turnover time is τr = 10s. Blue curve indicates average strain on all extended filaments; red curve indicates average strain on all compressed filaments; yellow curve indicates average strain on all filaments. (PDF) [file pcbi.1005811.s008.pdf]
